# Study on the host–guest interactions between tetramethyl cucurbit[6]uril and 2-heterocyclic-substituted benzimidazoles†

**DOI:** 10.1039/d3ra07810h

**Published:** 2024-01-16

**Authors:** Yanan Ye, Peihua Ma, Yue Ma, Naqin Yang, Xiaoqian Chen, Xinan Yang, Lingyi Shen, Xin Xiao

**Affiliations:** a Key Laboratory of Macrocyclic and Supramolecular Chemistry of Guizhou Province, Institute of Applied Chemistry, Guizhou University Guiyang 550025 China phma@gzu.edu.cn; b Guiyang College of Humanities and Science Guiyang 550025 China; c School of Basic Medicine, Guizhou Medical University Guiyang 550025 China

## Abstract

Cucurbit[*n*]urils (Q[*n*]s) are a class of supramolecular host compounds with hydrophilic carbonyl ports and hydrophobic cavities, which can selectively form host–guest inclusion complexes with guest molecules to change the properties of guest molecules. In this paper, tetramethyl cucurbit[6]uril (TMeQ[6]) was used as the host and three 2-heterocyclic substituted benzimidazole derivatives as the guests, and their modes of interaction were investigated using X-ray crystallography, ^1^H NMR spectrometry, and other analytical techniques. The results showed that TMeQ[6] formed a 1 : 1 host–guest inclusion complex with three guest molecules, and the binding process between them was mainly enthalpy-driven. The X-ray diffraction analysis indicated that the main driving forces for the formation of these three inclusion complexes included hydrogen bonding interactions and ion dipole interactions. There are two modes of interaction between G3 and TMeQ[6] in the liquid phase, indicating that the benzimidazole ring and heterocyclic substituents on the guest molecule compete with the cavity of TMeQ[6]. Besides, the addition of TMeQ[6] significantly enhanced the fluorescence of these guests and slightly improved their solubility.

## Abstract 2

The different interaction modes between TMeQ[6] and the guests indicate that the benzimidazole ring and heterocyclic substituted groups of the guest molecules are in competition with the cavity of TMeQ[6].

## Graphical abstract

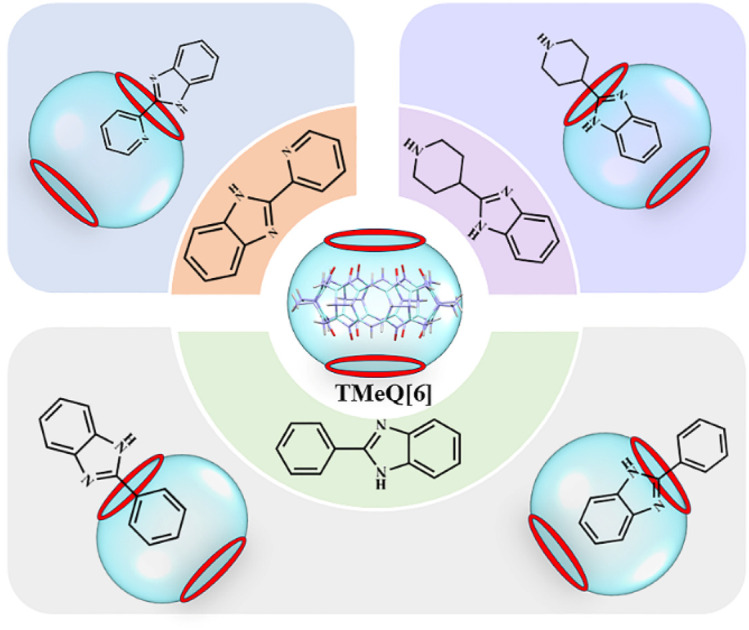

## 1. Introduction

The investigation of host–guest chemistry has emerged as a significant area of study within supramolecular chemistry. The utilization of the host–guest interaction between guest molecules and macrocycles presents an opportunity to fabricate supramolecular assemblies characterized by unique architectures, enduring characteristics, and versatile functionalities. Cucurbit[*n*]urils (Q[*n*]s, *n* = 5–8, 10, and 13–15)^1–6^ are a class of representative host molecules in macrocycles, which have a rigid structure composed of *n* glycoluril units and exhibit excellent stability. The hydrophobic effect of the cavity and the negative charge of the port are two essential characteristics of Q[*n*]s that aid in forming various host–guest compounds due to their exceptional selectivity and affinity for particular molecules.^7–11^ These unique properties of Q[*n*]s have led to their widespread use in ion recognition,^12,13^ supramolecular catalysis,^14,15^ drug carriers,^16,17^ and luminescent materials.^18,19^ However, Q[6, 8, 10] have inferior solubility due to their highly symmetrical spatial structure, which can be improved and modified by introducing substituents on the waist of the Q[*n*]s. A series of modified cucurbit[*n*]urils have been reported, such as methyl, hydroxyl, cyclopentyl, and cyclohexyl substituted Q[*n*]s.^20–24^ Tetramethyl cucurbit[6]uril (TMeQ[6]) exhibits superior water solubility and greater affinity compared to Q[6].^25^ Additionally, TMeQ[6] possesses a unique elliptical cavity that enables the formation of stable host–guest inclusion complexes with various guest molecules, including aniline derivatives, viologen derivatives, anti-tuberculosis drugs, and others.^26–28^

**Fig. 1:**
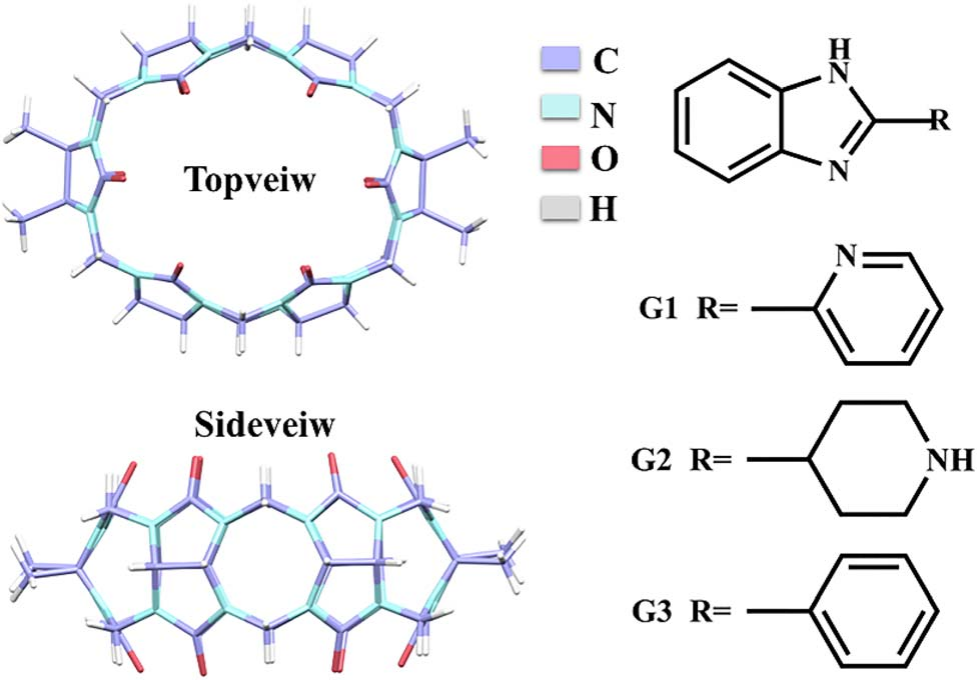
Structural formula of TMeQ[6], 2-(2-pyridyl)benzimidazole (G1), 2-(4-piperidyl) benzimidazole (G2), and 2-phenylbenzimidazole (G3).

Benzimidazole derivatives constitute a class of benzo-heterocyclic compounds that possess notable biological activity and pharmacological effects.^29^ These compounds find extensive utilization in pharmaceutical and agricultural domains for their advantageous characteristics such as high stability and low toxicity. Specifically, they are employed in various applications including antibacterial, anti-inflammatory, antitumor, insecticidal, and herbicidal.^30–33^ Nevertheless, their bioavailability is severely constrained by limited water solubility. It is known that the encapsulation of drug molecules using Q[*n*]s can improve the solubility of the guest,^34–37^ we expected to use TMeQ[6] as a drug carrier for benzimidazole drug molecules to encapsulate the drug in order to augment its solubility, prolong the release time, enhance the efficacy, and achieve other desirable outcomes. Hence, it is of utmost significance to investigate the interactions between Q[*n*]s and benzimidazole derivatives in the context of host–guest chemistry. According to reports, benzimidazole derivatives can generate stable host–guest complexes with Q[*n*]s (*n* = 6–8), cyclohexanocucurbit[6]uril(Cy6Q[6]), dicyclohexanocucurbit[6]uril (CyH2Q[6]), and others.^38–40^ This study employed various analytical techniques, including ^1^H NMR spectroscopy, UV-vis absorption spectroscopy, fluorescence analysis, mass spectrometry, isothermal titration calorimetry (ITC), and single crystal X-ray diffraction to investigate the interaction modes and spectroscopic characteristics of TMeQ[6] with three 2-heterocyclic-substituted benzimidazoles: 2-(2-pyridyl)benzimidazole (G1), 2-(4-piperidyl)benzimidazole (G2), and 2-phenylbenzimidazole (G3) (Fig. 1). Additionally, the study examined the solubilization effect of TMeQ[6] on these three guest compounds.

## 2. Experimental section

### 2.1 General materials

TMeQ[6] was synthesized according to a method in the literature.^25^ The guests (G1–G3) were purchased from Aladdin Industrial Corporation (AR, Shanghai, China) and used without any further purification.

The procedure for synthesising complexes is elaborated upon in the ESI.† Crystallographic data reported in this manuscript have been deposited with Cambridge Crystallographic Data Centre as supplementary publication no. CCDC-2259636 (complex 1), CCDC-2280674 (complex 2), and CCDC-2278652 (complex 3). Copies of the data can be obtained free of charge *via* CCDC Website. Instruction for depositing the crystallographic data is available on the web at https://www.ccdc.cam.ac.uk/conts/depositing.html.

### 2.2 Experimental methods

All ^1^H NMR spectroscopy data were measured and recorded in D_2_O (pD = 2.0, adjusted with deuterated hydrochloric acid) at 293 K on a JEOL JNM-ECZ 400 s NMR spectrometer.^12^

UV-vis absorption and fluorescence spectroscopy were measured on a UV-2700 dual-beam UV-vis spectrophotometer and Varian Carye-Clipse fluorescence spectrophotometer, respectively.

Using an Agilent 6545B instrument, high-performance liquid chromatography-quadrupole time-of-flight mass spectrometry (HPLC-QTOF-MS) data of the inclusion complexes were collected.

The thermodynamic properties of each system were assessed with a nano ITC isothermal calorimetric titrator, while the measurement and data processing techniques adhered to a previously established methodology found in the literature.^41^

Solubility experiments were conducted using a method reported in the literature.^42^

## 3. Results and discussions

### 3.1 ^1^H NMR spectroscopic analysis

**Fig. 2:**
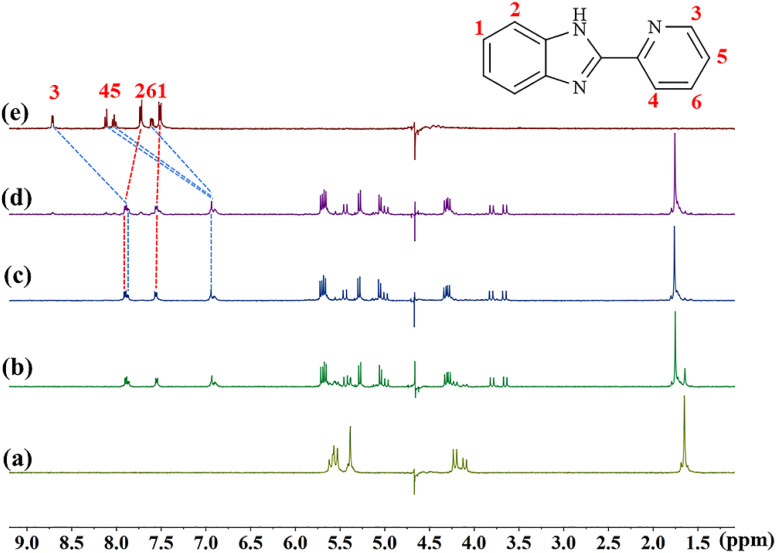
^1^H NMR spectra obtained for the interaction between TMeQ[6] and G1 (25 °C, 400 MHz) (D_2_O, pD = 2) in the presence of TMeQ[6] (0.5 mM) and (a) 0, (b) 0.8, (c) 1.0, and (d) 1.2 equiv. of G1 and (e) pure G1.

The host–guest interaction modes of TMeQ[6] with (G1–G3) were investigated using ^1^H NMR spectroscopy. Fig. 2 is the ^1^H NMR titration spectra of TMeQ[6] interacting with different equivalents of G1. Upon the addition of G1, when the molar ratio of TMeQ[6] to G1 was 1 : 1, the H_3_, H_4_, H_5_ and H_6_ peaks corresponding to the pyridine group shift upfield by 0.85, 1.17, 1.09, and 0.67 ppm, respectively, due to the shielding effect of the hydrophobic cavity of TMeQ[6] and the pyridine group entering the cavity of TMeQ[6]. On the contrary, the H_1_ and H_2_ protons on the benzimidazole ring shift downfield by 0.04 and 0.17 ppm, respectively, due to the deshielding effect of the portal of TMeQ[6] and the benzimidazole ring being located at the port of TMeQ[6]. When the amount of G1 was further increased, a free peak appeared, which indicated that TMeQ[6] and G1 formed a 1 : 1 host–guest inclusion complex. It can also be concluded from the ^1^H NMR titration spectra of TMeQ[6] and G2–G3 that TMeQ[6] and G2–G3 can form 1 : 1 host–guest inclusions (Fig. S1 and S2†), respectively. The difference is that benzimidazole part of G2 into the cavity of TMeQ[6]. Interestingly, G3 has two modes of interaction, the substituent and the benzimidazole ring can alternatively enter the cavity of TMeQ[6]. A detailed ^1^H NMR description of G2 and G3 was given in the ESI.†

### 3.2 UV-vis absorption and fluorescence spectroscopy analysis

**Fig. 3:**
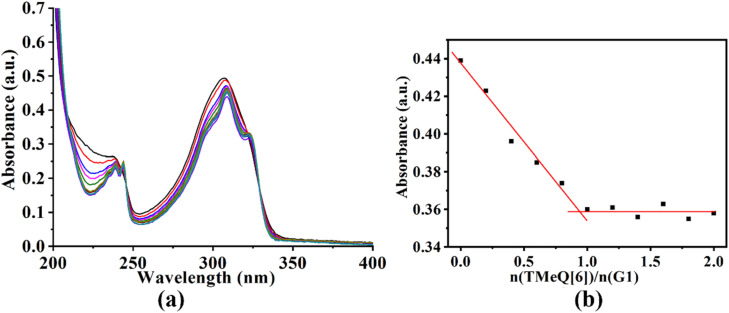
(a) The UV-vis spectra obtained upon adding TMeQ[6] (0, 0.2, 0.4,… 1.6, 1.8, and 2.0, equiv.) to guest G1 (1 × 10^−3^ mol L^−1^, pH = 2). (b) Plots of *n*(TMeQ[6])/*n*(G1) *vs.* the UV-vis absorption of G1.

To gain additional insight into the mode of interaction, the UV-vis absorption and fluorescence spectra of TMeQ[6] and G1–G3 were examined. Adding TMeQ[6] to the guest solution elicited changes to the absorbance and fluorescence intensity of the solution. The UV-vis spectra of G1 (Fig. 3) shows that G1 had a significant absorption peak at 308 nm. The absorbance of G1 was steadily reduced as the TMeQ[6] content in the solution increased. Once *n*(TMeQ[6])/*n*(G1) = 1, the absorbance became stable. The host–guest binding ratio of TMeQ[6] and G1 was ∼1 : 1 upon fitting the data.

**Fig. 4:**
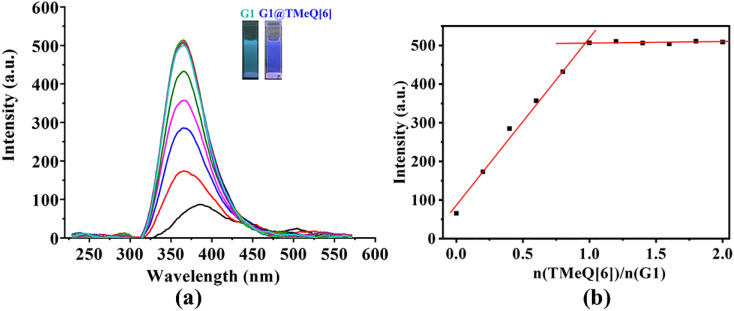
(a) The fluorescence spectra obtained upon adding TMeQ[6] (0, 0.2, 0.4,… 1.6, 1.8, and 2.0, equiv.) to guest G1 (1 × 10^−3^ mol L^−1^) (pH = 2). (b) Plots of *n*(TMeQ[6])/*n*(G1) *vs.* the fluorescence intensity of G1; insert: photographs of G1 and G1@TMeQ[6] under UV light irradiation (302 nm).

G1 only displays a faint fluorescence at 387 nm, as shown in Fig. 4. The fluorescence intensity of G1 shows an ongoing increase with the addition of TMeQ[6] (Fig. 4 insert). When exposed to UV radiation, the color changed from marine blue to blue. It was hypothesized that TMeQ[6] and G1 formed an inclusion complex, which alters the electron cloud density of the guest molecule. The fluorescence intensity smoothed off when the ratio of *n*(TMeQ[6])/*n*(G1) was 1 : 1. Furthermore, it was deduced *via* data fitting that the binding ratio between TMeQ[6] and G1 was 1 : 1. The UV-vis and fluorescence spectra obtained for TMeQ[6]-(G2–G3) were comparable to the phenomenon of TMeQ[6]-G1 (Fig. S3–S6†), demonstrating that the interaction ratio of TMeQ[6] with (G2–G3) was also 1 : 1. In addition, the addition of TMeQ[6] significantly enhanced the fluorescence of these guests, indicating that new fluorescent probes based on the interaction between TMeQ[6] and 2-heterocyclic-substituted benzimidazoles can be constructed, which has application prospects in the field of molecular recognition and detection.

### 3.3 HPLC-QTOF mass spectrometry analysis

The TMeQ[6] and (G1–G3) host–guest interactions were further investigated using mass spectrometry, as shown in Fig. S7–S9,† in which the molecular peaks were observed at *m*/*z* 1248.4439 ([TMeQ[6]+G1]^+^, calculated: 1248.0777), 1254.4915 ([TMeQ[6]+G2]^+^, calculated: 1254.1962), and 1247.4494 ([TMeQ[6]+G3]^+^, calculated: 1247.0896), as distinct signal peaks. These data more intuitively illustrate that TMeQ[6] and G1–G3 all form 1 : 1 host–guest inclusion complexes, which was consistent with our previous analysis.

### 3.4 Isothermal titration calorimetry analysis

ITC was implemented to investigate the thermodynamic properties and binding constants of complex (1–3) (Fig. S10–S12, in the ESI† for this research). Our results demonstrated that the *K*_a_ values of TMeQ[6] interacting with (G1–G3) were in the range of 4.051 × 10^5^–6, 639 × 10^5^ L mol^−1^, and the Δ*G* values were in the range of −33.23–−32.01 kJ mol^−1^ as Table 1. These results indicate that the three benzimidazole derivatives can effectively bind to TMeQ[6] and the encapsulants had outstanding thermal stability and binding strength. In addition, according to the enthalpy and entropy change values of the three reactions, it could be inferred from the Gibbs free energy (Δ*G* = −RT ln*K*_a_ = Δ*H* − *T*Δ*S*), indicating that the three systems were exothermic and mainly enthalpy driven.

**Table 1:** Thermodynamic parameters obtained for the interactions formed between TMeQ[6] and guest molecules G1–G3

Complex	*K*_a_ (L mol^−1^)	Δ*H* (kJ mol^−1^)	−*T*Δ*S* (kJ mol^−1^)	Δ*G* (kJ mol^−1^)
1	6.639 × 10^5^	−88.96	55.72	−33.23
2	4.051 × 10^5^	−31.40	−0.604	−32.01
3	5.552 × 10^5^	−32.87	7.818 × 10^−2^	−32.79

### 3.5 Solubility analysis

We used the solubility method to test whether guest molecules (G1–G3) have solubilizing effects on guest molecules when interacting with TMeQ[6]. Fig. S13–S15† show the standard calibration curves and regression equations obtained for G1–G3 and (G1–G3)@TMeQ[6]. The calculated solubilities of the TMeQ[6]-(G1–G3) system inclusion complexes in an aqueous solution were 1.48 × 10^−4^, 4.51 × 10^−4^, and 4.13 × 10^−5^ mol L^−1^, respectively. When compared with the solubility of the pure guest molecules (G1: 1.28 × 10^−4^ mol L^−1^, G2: 3.47 × 10^−4^ mol L^−1^, and G3: 3.92 × 10^−5^ mol L^−1^), the solubilities of the host–guest inclusion complexes were ∼20% higher.

### 3.6 Crystal structure description

**Fig. 5:**
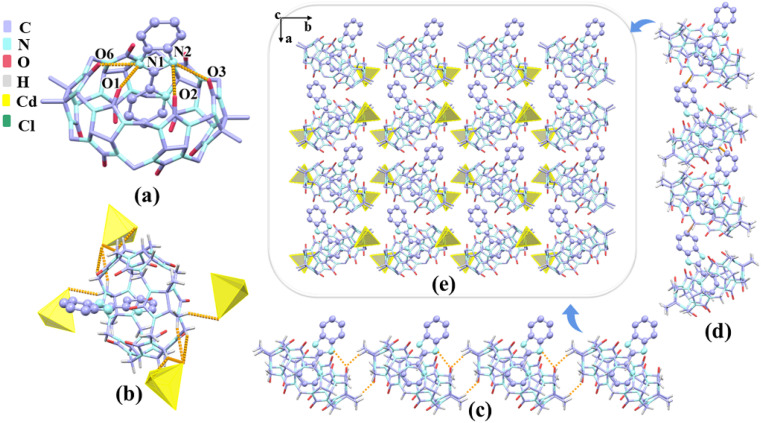
(a) The binding mode between G1 and TMeQ[6] (all hydrogen atoms and equilibrium charges are omitted). (b) One-dimensional supramolecular chain of 1. (b) The detailed ion–dipole interaction formed between complex 1 and [CdCl_4_]^2−^. (c and d) One-dimensional supramolecular chains of complex 1. (e) The stacking diagram of complex 1 observed along the *c*-axis.

In the presence of [ZnCl_4_]^2−^ or [CdCl_4_]^2−^ anions as a structure-directing agent, we obtained single crystals of complexes 1–3. The crystals of complex 1 shows a monoclinic *P*2_1_/*c* space group as Table S2.†Fig. 5a shows the protonated G1 was fixed at the port of TMeQ[6] *via* N(1)–H⋯O(1), N(1)–H⋯O(6), N(2)–H⋯O(2), N(2)–H⋯O(3) hydrogen bonds formed between the nitrogen atoms (N1, N2) on the imidazole ring and the carbonyl oxygen atoms (O1, O2, O3, and O6) of TMeQ[6]. The bond lengths were 2.777, 2.769, 2.967, and 2.773 Å, respectively. The substituent entered the cavity of TMeQ[6] with an interaction ratio of 1 : 1, which was in agreement with ^1^H NMR spectrometry results. Fig. 5c and d show the two one-dimensional supramolecular chains of complex 1 consist of C–H⋯O hydrogen bonding interactions and C–H⋯π interactions, respectively. Fig. 5b shows each TMeQ[6] was coupled with four [CdCl_4_]^2−^*via* C–H⋯Cl ion–dipole interactions, resulting in the formation of an ordered supramolecular framework structure (Fig. 5e).

**Fig. 6:**
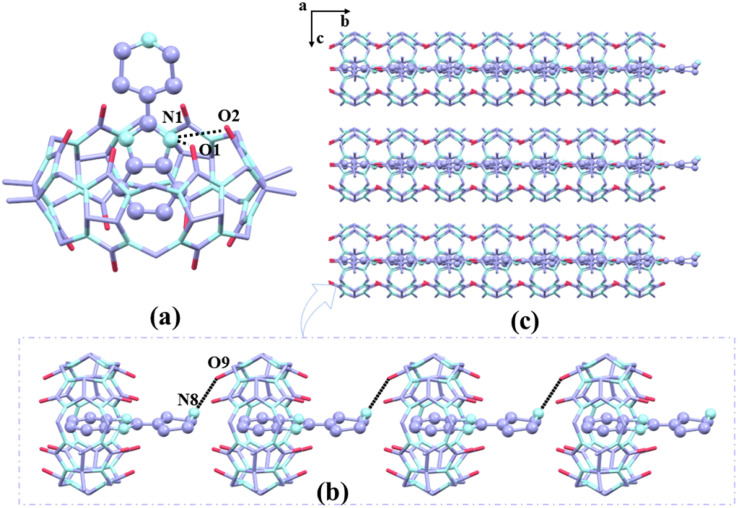
(a) The binding mode between G2 and TMeQ[6] (all hydrogen atoms and equilibrium charges are omitted). (b) One-dimensional supramolecular chains of complex 2. (c) The stacking diagram of complex 2 observed along the *a*-axis.

The crystals of complex 2 exhibit a monoclinic crystal system with a space group of *C*2 (Table S2†). According to Fig. 6a, the piperidinyl group was located at the port of TMeQ[6] and the benzimidazole ring of G2 entered the cavity of TMeQ[6]. The nitrogen atoms on the benzimidazole ring and the carbonyl oxygens of TMeQ[6] interact *via* N(1)–H⋯O(1) and N(1)–H⋯O(2) hydrogen bonds to form the inclusion complex with bond lengths of 2.933 and 2.741 Å, respectively (Fig. 6b). The nitrogen atoms on the piperidine group (N8) were connected to the carbonyl oxygen of the neighboring guanine ring *via* N(8)–H⋯O(9), forming a one-dimensional supramolecular chain with G2 functioning as a bridge and an ordered two-dimensional lamellar supramolecular structure along the a-axis (Fig. 6c).

**Fig. 7:**
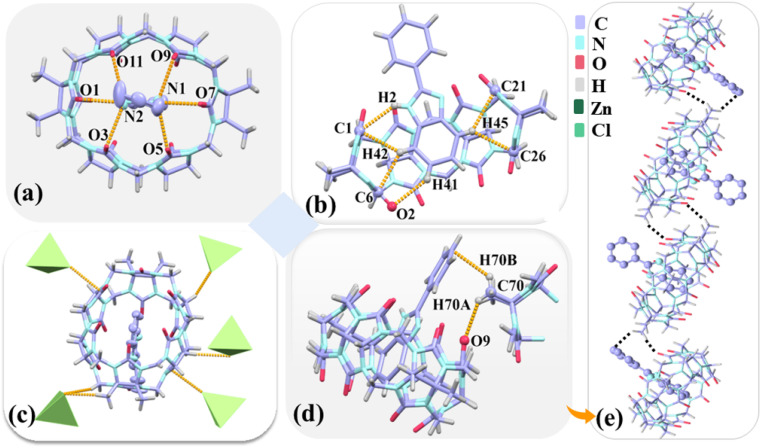
(a and b) Hydrogen-bonding interactions formed between G3 and TMeQ[6]. (c) Detailed ion–dipole interaction formed between 3 and [ZnCl_4_]^2−^ anions. (d) Hydrogen bond and dipole interactions of adjacent complex 3. (e) One-dimensional supramolecular chains of complex 3.

Complex 3 belongs to the triclinic *P*1̄ space group (Table S2†). Fig. 7 shows the protonated G3 forms a 1 : 1 host–guest inclusion with TMeQ[6], which corresponds to mode A observed in the ^1^H NMR spectrometry results obtained for G3. Hydrogen bonds exist between the nitrogen atoms (N1 and N2) on the imidazole ring of G3 and the port carbonyl oxygen atoms of TMeQ[6] as N(1)–H⋯O(5), N(1)–H⋯O(7), N(1)–H⋯O(9), N(2)–H⋯O(1), N(2)–H⋯O(3), and N(2)–H⋯O(11) with bond lengths of 2.991, 2.734, 2.956, 2.786, 2.946, and 3.017 Å, respectively(Fig. 7a). G3 and TMeQ[6] were anchored in the cavity of TMeQ[6] *via* C(1)–H(2)⋯N, C(1)–H(42)⋯C, and C(6)–H(42)⋯C interactions (Fig. 7b). The outer surface of each TMeQ[6] was enriched with eight [ZnCl_4_]^2−^*via* C–H⋯Cl ion–dipole interactions (Fig. 7c). The two inclusion complexes were linked to form a one-dimensional supramolecular chain *via* C(70)–H(70A)⋯O(9) hydrogen bond and C(70)–H(70A)⋯πinteraction (Fig. 7d and e).

## 4. Conclusion

The present study aimed to evaluate the interaction mechanisms of TMeQ[6] with three 2-substituted benzimidazole derivatives in the solid–liquid state through the use of diverse characterisation approaches. The findings suggest that within the solid state, the three guests form 1 : 1 host–guest inclusion complexes with TMeQ[6] respectively. Pyridine group exhibits the higher propensity for binding with the cavity of TMeQ[6] than the benzimidazole ring. It is shown that the pyridine group of G1 entered the cavity of TMeQ[6], while benzimidazole ring of G2 and G3 did. Three new supramolecular assemblies are formed *via* the synergistic effects of hydrogen bonds, ion–dipole interactions, and C–H⋯π interactions. In the liquid state, the encapsulation of the guest molecule by TMeQ[6] significantly enhanced the fluorescence intensity of the guest. The main driving force of these three systems was enthalpy-driven. G1 and G2 were consistent with the interaction mode observed in the solid state, whereas G3 had two modes of interaction (A and B) due to the competing interactions of the benzimidazole ring and substituents in the molecule with the TMeQ[6] cavity. Mode A matched the crystal structure, while mode B was the entry of phenyl group into the cavity of TMeQ[6]. Furthermore, the inclusion complexes formed by TMeQ[6] and G1–G3 have solubilizing impact on the guest molecules. This study provides a theoretical basis for investigating the solubility and stability of drug molecules. Our results provide a reference for the application of Q[*n*]s in drug encapsulation, delivery, and molecular recognition.

## Data availability

Data will be made available on request.

## Author contributions

Yanan Ye: conceptualization, software, data curation, writing–original draft. Peihuahua Ma: review & editing, formal analysis, supervision, resources. Yue Ma: resources, Naqin Yang: resources, methodology, writing–review & editing. Xiaoqian Chen: visualization, resources. Xinan Yang: writing–review & editing, data curation. Lingyi Shen: validation, visualization, Xin Xiao formal analysis, supervision.

## Conflicts of interest

The authors declare that they have no known competing financial interests or personal relationships that could have appeared to influence the work reported in this paper.

## Supplementary Material

RA-014-D3RA07810H-s001

RA-014-D3RA07810H-s002
