# Supplementary material for: Study on the host–guest interactions between tetramethyl cucurbit[6]uril and 2-heterocyclic-substituted benzimidazoles
Source: RSC Adv. 2024 Jan 16;14(4):2652–8. doi: 10.1039/d3ra07810h (PMC10790279; doi:10.1039/d3ra07810h)
Supplement: RA-014-D3RA07810H-s001 [file RA-014-D3RA07810H-s001.pdf]

Electronic Supplementary Material (ESI) for RSC Advances.
This journal is © The Royal Society of Chemistry 2024

Support information

Study on the Host-Guest Interactions Between Tetramethyl Cucurbit[6]uril and 2-Heterocyclic-Substituted Benzimidazoles

Yanan Ye [a], Peihua Ma [a*], Yue Ma [b], Naqin Yang [a], Xiaoqian Chen [a], Xinan Yang [a], Lingyi Shen [c], Xin Xiao [a]

[a] Key Laboratory of Macrocyclic and Supramolecular Chemistry of Guizhou Province, Institute of Applied Chemistry, Guizhou University, Guiyang 550025, China.

[b] Guiyang College of Humanities and Science, Guiyang 550025, China.

[c] School of Basic Medicine, Guizhou Medical University, Guiyang 550025, China.

[a*] E-mail: phma@gzu.edu.cn

1.Calculation of binding constants

Table S1 The binding constants of the inclusion complexes calculated under three experimental methods

Complex	ITC Ka($L\cdot mol^{-1}$)	UV-Vis titration Ka ($L\cdot mol^{-1}$)	Fluorescence titration Ka ($L\cdot mol^{-1}$)
**1**	6.639×10^5	2.182×10^5	2.646×10^5
**2**	4.051×10^5	1.879×10^5	1.47×10^5
**3**	5.552×10^5	2.441×10^5	1.031×10^5

Based on the Benesi Hildebrand method [1], the binding constants of the inclusion complexes were calculated using UV-Vis spectroscopy and fluorescence titration. Compared with the binding constants determined by ITC, the binding coefficients determined by UV-Vis and fluorescence titration were smaller(Table S1). It is

considered that this is due to that there are more factors affecting the experimental results in the process of UV-Vis spectroscopy and fluorescence measurement than in ITC. The ITC theoretical basis of the system was the thermal effect generated during the mixing process of host guest solutions, and the binding constant was obtained through computer processing, with higher accuracy. Although the calculation results of the binding constants of the three inclusion complexes by the three methods were not completely consistent, the larger binding constants indicated that the three benzimidazole derivatives formed stable inclusion complexes with TMeQ[6].

2. Synthesis of complex

Synthesis of complex **1**: TMeQ[6] (15 mg, 14.25 μmol) was dissolved in hydrochloric acid solution (5 mL, 3 mol/L). $CdCl_2$ (5.0 mg, 27.27 μmol) and G1 (5.0 mg, 25.61 μmol) were added to the resulting mixture. The mixture was then subjected to ultrasonic oscillation, followed by heating at 80°C for a duration of 10 minutes. Subsequently, the mixture was allowed to stand at room temperature for a period of 7–14 days. This process resulted in the formation of complex **1** with a yield of 38%.

Synthesis of complex **2**: TMeQ[6] (15 mg, 14.25 μmol) was dissolved in hydrochloric acid solution (5 mL, 3 mol/L). G2 (5.0 mg, 25.84 μmol) were added to the resulting mixture. The mixture was then subjected to ultrasonic oscillation, followed by heating at 80°C for a duration of 10 minutes. Subsequently, the mixture was allowed to stand at room temperature for a period of 7–14 days. This process resulted in the formation of complex **2** with a yield of 28%.

Synthesis of complex **3**: TMeQ[6] (15 mg, 14.25 μmol) was dissolved in hydrochloric acid solution (5 mL, 3 mol/L). $ZnCl_2$ (5.0 mg, 36.68 μmol) and G3 (5.0 mg, 25.74 μmol) were added to the resulting mixture. The mixture was then subjected to ultrasonic oscillation, followed by heating at 80°C for a duration of 10 minutes. Subsequently, the mixture was allowed to stand at room temperature for a period of 7–

14 days. This process resulted in the formation of complex **3** with a yield of 35%.

3. ^{1}H NMR spectroscopic analysis

The ^{1}H NMR titration spectra obtained for TMeQ[6] in the presence of different equivalents of G2 are displayed in Figure 1. The benzimidazole part was shielded and enters the cavity of TMeQ[6] when the molar ratio was 1:1, the H_1 and H_2 peaks of G2 shift upfield by 0.76 and 0.78 ppm, respectively, when compared to the free state, while H_3, H_{4a}, H_{4b}, H_{5a}, and H_{5b} shift downfield by 0.03, 0.32, 0.03, 0.35, and 0.32 ppm, respectively, indicating that the piperidinyl group was deshielded by TMeQ[6] and positioned at the port of TMeQ[6]. The proton signal presents a free peak as the G1 concentration was further raised, which was evidence that TMeQ[6] and G2 form a 1:1 host-guest inclusion complex.

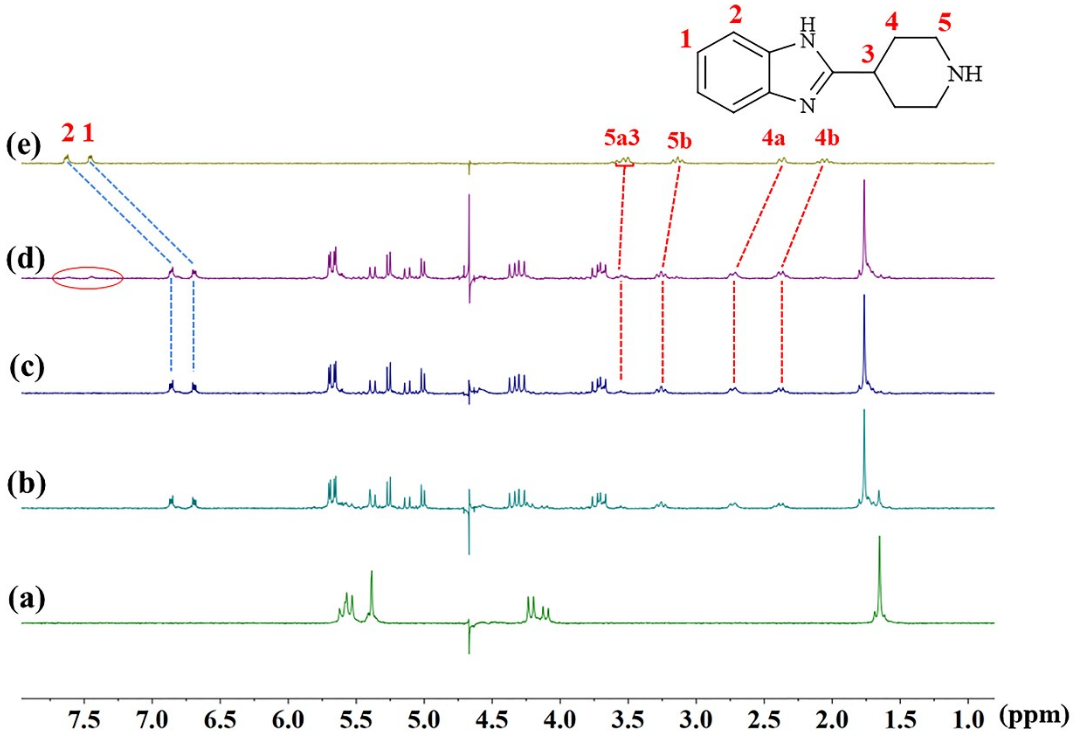

Figure S1. ^{1}H NMR spectra obtained for the interaction between TMeQ[6] and G2 (25°C, 400 MHz) (D_2O, pD =2):in the presence of TMeQ[6] (0.5mM) and (a) 0, (b) 0.8, (c) 1.0, and (d) 1.1 equiv. of G2, and (e) pure G2.

The ^{1}H NMR titration spectra obtained for TMeQ[6] in interaction with different equivalents of G3 are shown in Figure S2. All of the proton peaks of G3 were split into

two groups, one of which moved to downfield and the other moved upfield, (the blue line signified mode **A**, whereas the red line signified mode **B**). This indicates that there are two modes of action between TMeQ[6] and G3. In mode **A**, the benzimidazole ring of G3 entered the cavity of TMeQ[6] and the phenyl group was situated at the port of TMeQ[6] when the molar ratio was 1:1. The H_{1a} and H_{2a} peaks of G3 were shifted upfield by 0.88 and 0.80 ppm, respectively, when compared with the free state, and H_{3a} and H_{4a} were shifted downfield by 0.37 and 0.20 ppm, respectively. However, mode **B** was the antithesis of mode **A**. H_{3b} and H_{4b} were shifted upfield by 1.32 and 0.57 ppm, respectively, when compared to the free state, while H_{1b} and H_{2b} were shifted downfield by 0.02 and 0.19 ppm, respectively. This demonstrated that TMeQ[6] and G3 form a 1:1 host-guest inclusion complex when the proton signal displayed a free peak as the concentration of G3 was further increased in both modes.

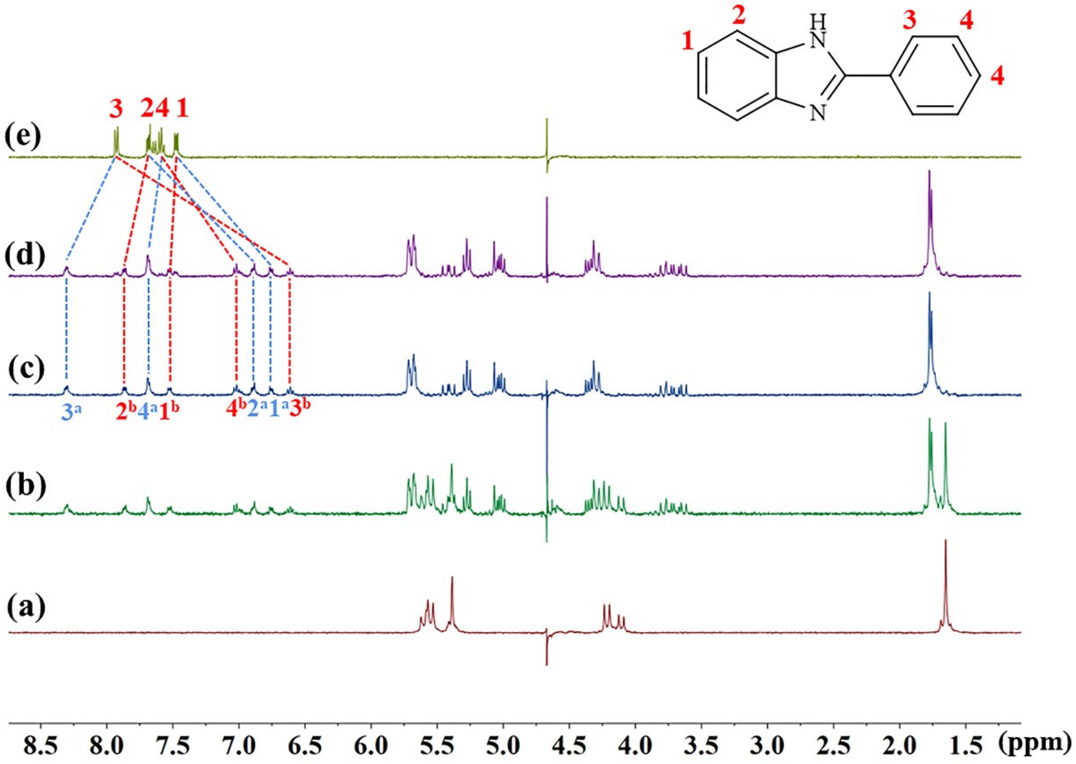

Figure S2. ^{1}H NMR spectra of the interaction between TMeQ[6] and G3 (25℃, 400 MHz) (D_2O, pD=2):in the presence of TMeQ[6] (0.5mM), the equivalent (a) 0, (b) 0.8, (c) 1.0 , (d)1.2, (e) pure G3

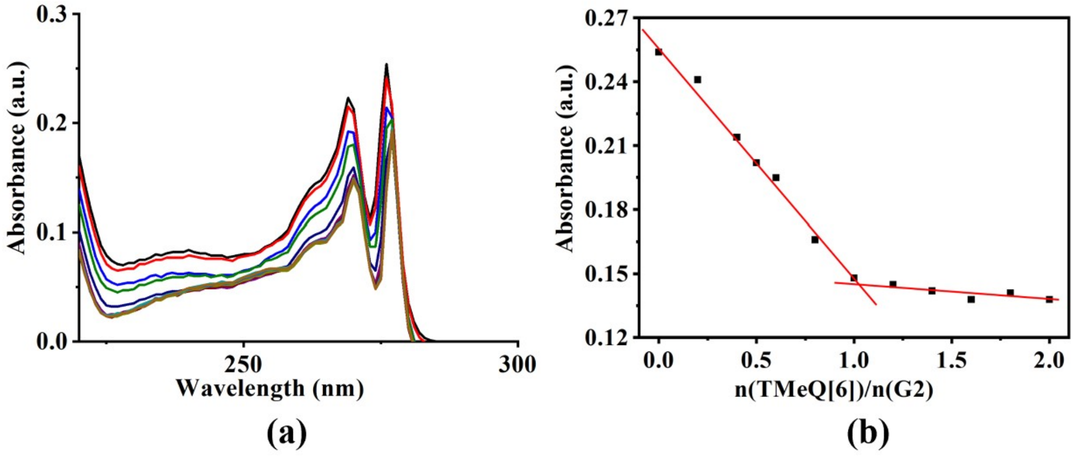

Figure S3. (a) The UV-Vis spectrogram of adding TMeQ[6] (0, 0.2, 0.4,…1.6, 1.8, 2.0, equivalent) to the guest G2 (1×10^{-3} mol L^{-1}, pH=2). (b) Plots of n(TMeQ[6])/n(G2) vs ultraviolet absorption of G2.

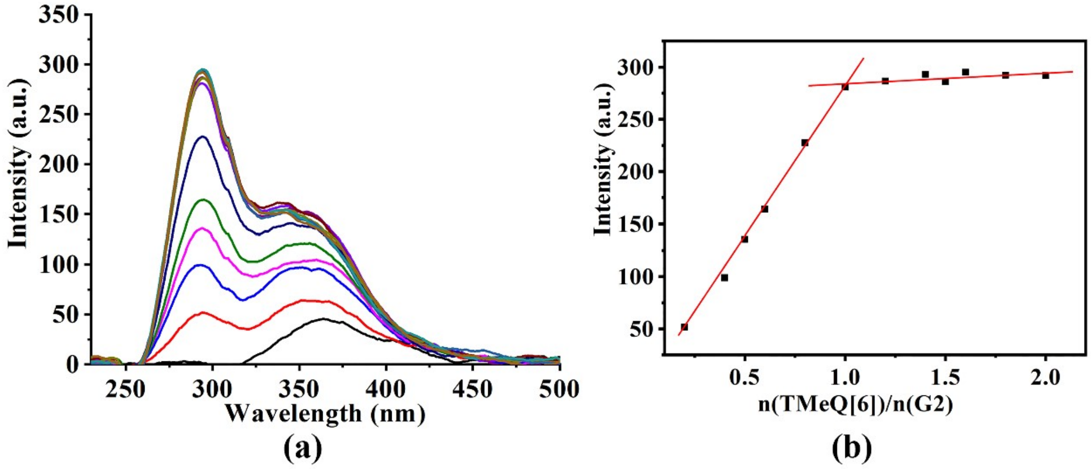

Figure S4. (a) The fluorescence spectra of adding TMeQ[6] (0, 0.2, 0.4,…1.6, 1.8, 2.0, equivalent) to the guest G2 (1×10^{-3} mol L^{-1}) (pH=2). (b) Plots of n(TMeQ[6])/n(G2) vs fluorescence intensity of G2.

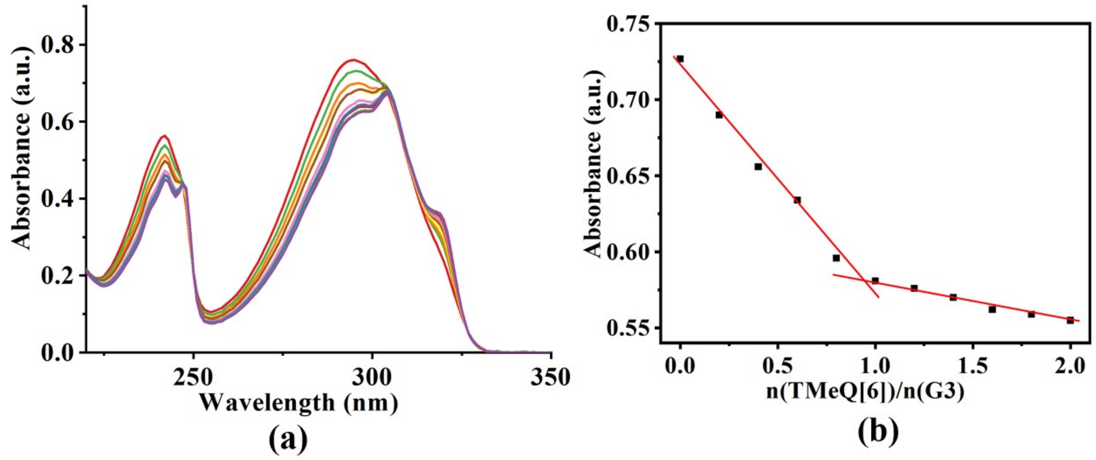

Figure S5. (a) The UV-Vis spectrogram of adding TMeQ[6] (0, 0.2, 0.4,…1.6, 1.8, 2.0, equivalent) to the guest G3 (1×10^{-3} mol L^{-1}, pH=2). (b) Plots of n(TMeQ[6])/n(G3) vs ultraviolet absorption of G3.

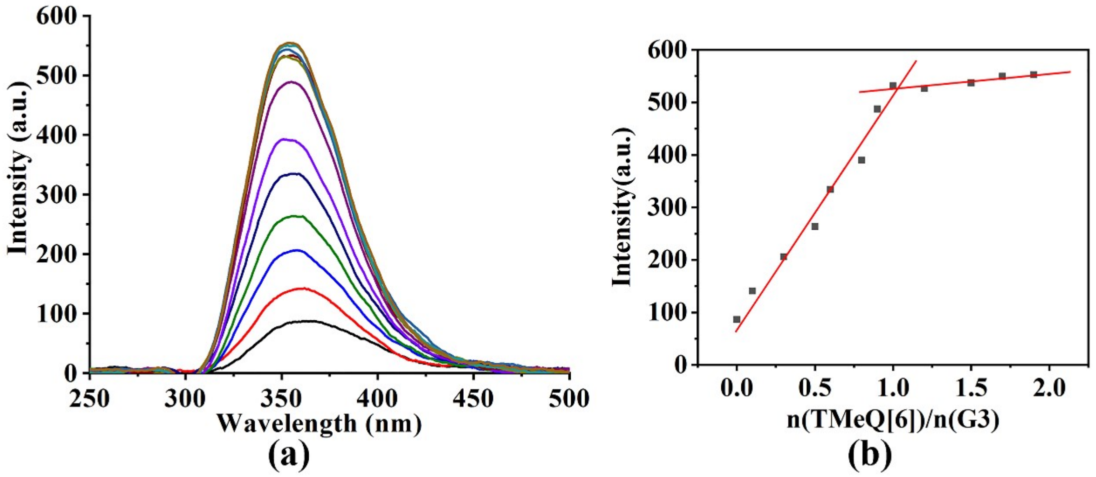

Figure S6. (a) The fluorescence spectra of adding TMeQ[6] (0, 0.2, 0.4,…1.6, 1.8, 2.0, equivalent) to the guest G3 (1×10^{-3} mol L^{-1}) (pH=2). (b) Plots of n(TMeQ[6])/n(G3) vs fluorescence intensity of G3.

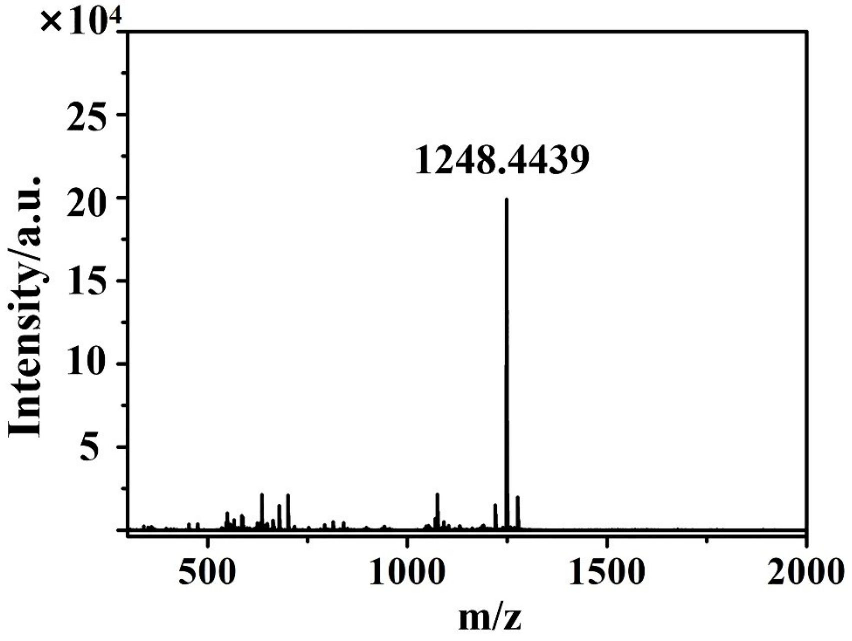

Figure S7. HPLC-QTOF mass spectra obtained for G1@TMeQ[6

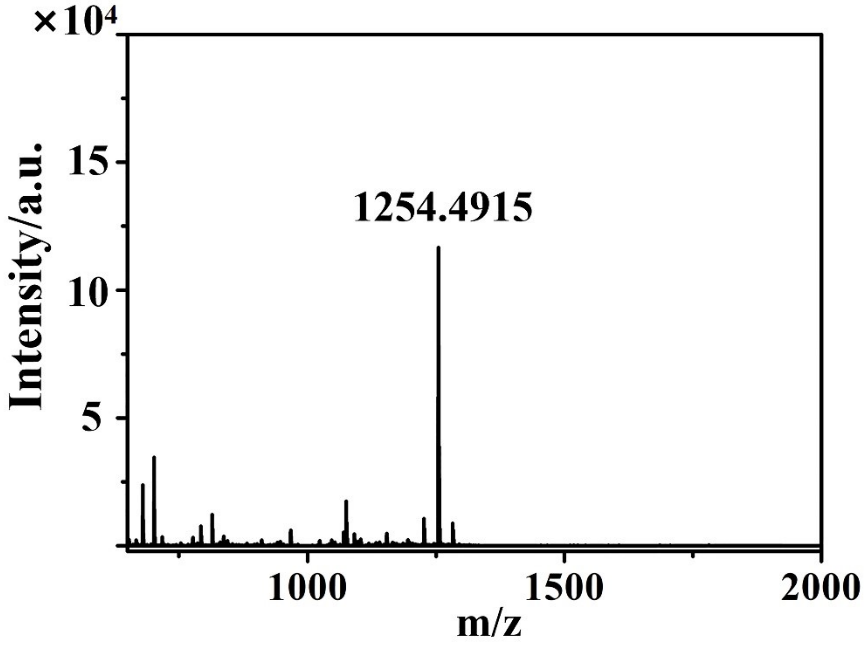

Figure S8. HPLC-QTOF mass spectra obtained for G2@TMeQ[6

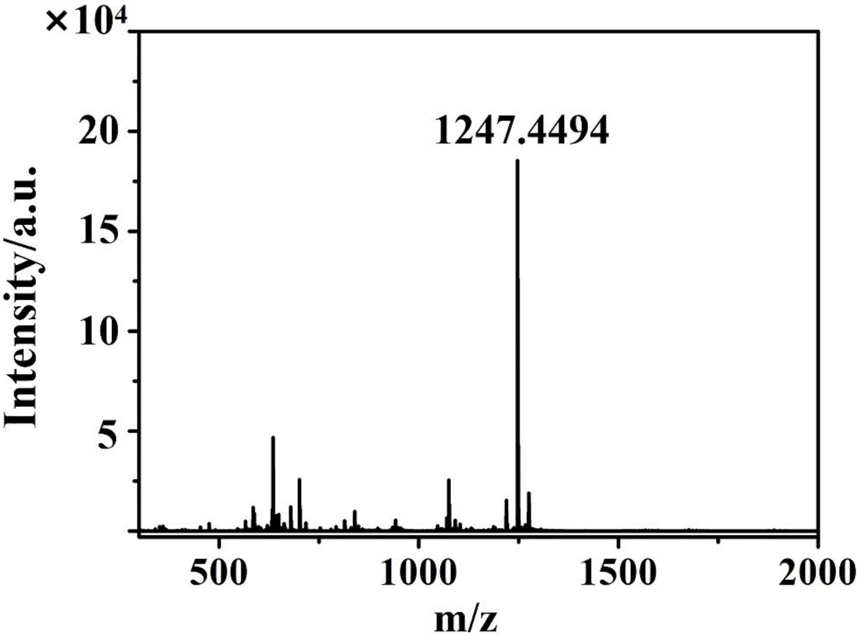

Figure S9. HPLC-QTOF mass spectra obtained for G3@TMeQ[6

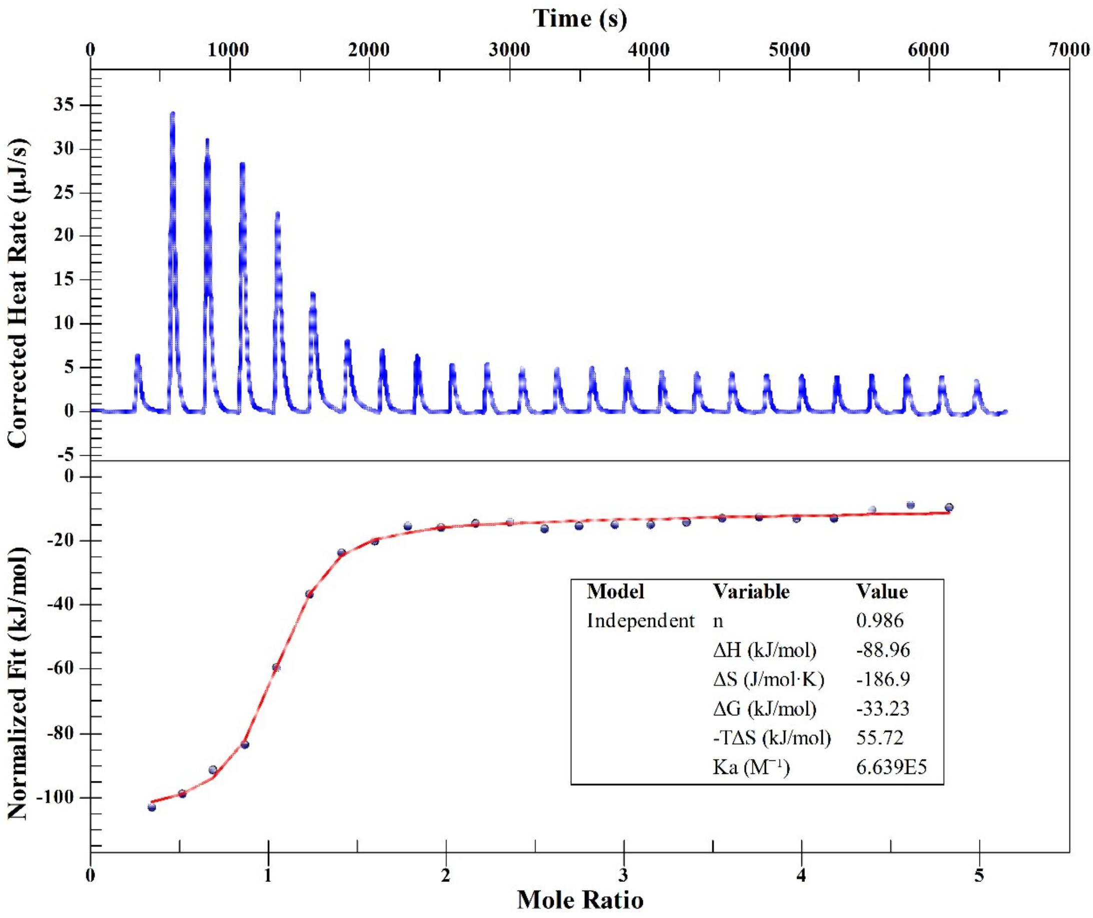

Figure S10. Titration diagram for isothermal titration calorimetry of G1@TMeQ[6].

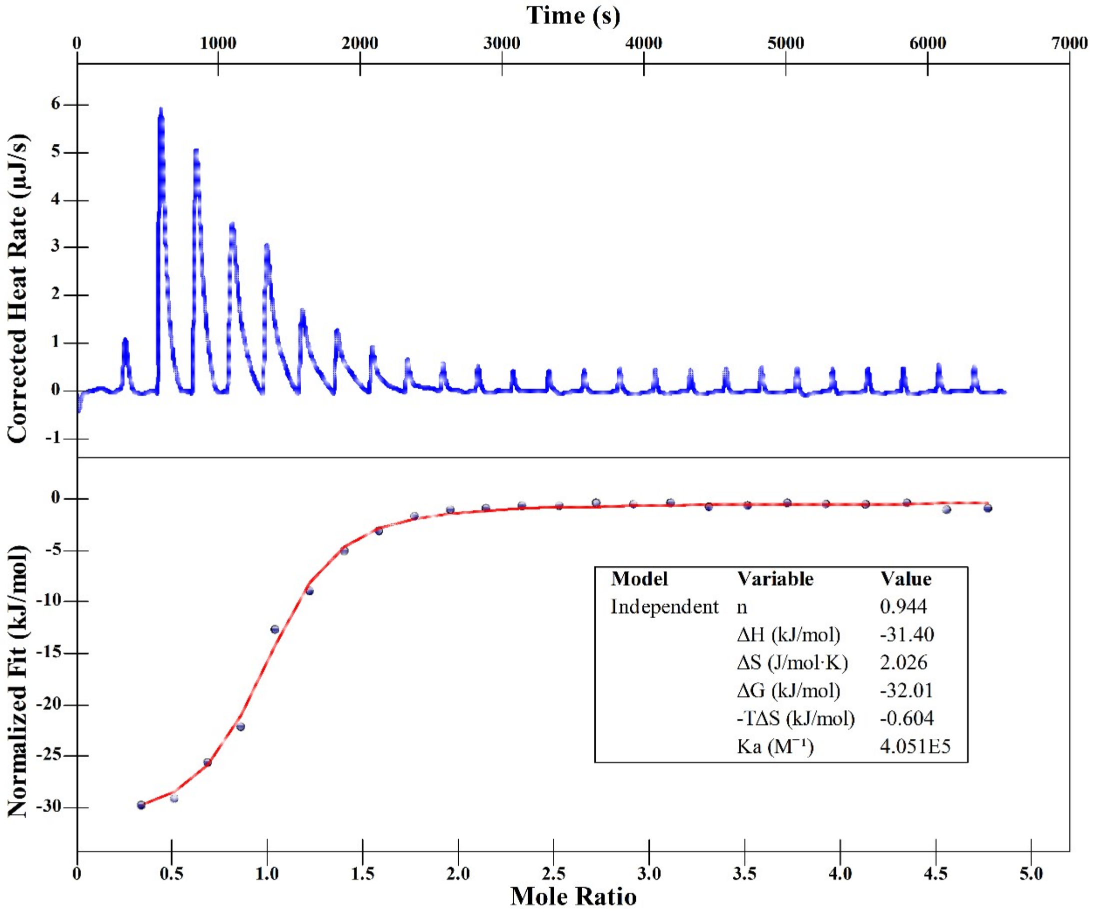

Figure S11. Titration diagram for isothermal titration calorimetry of G2@TMeQ[6].

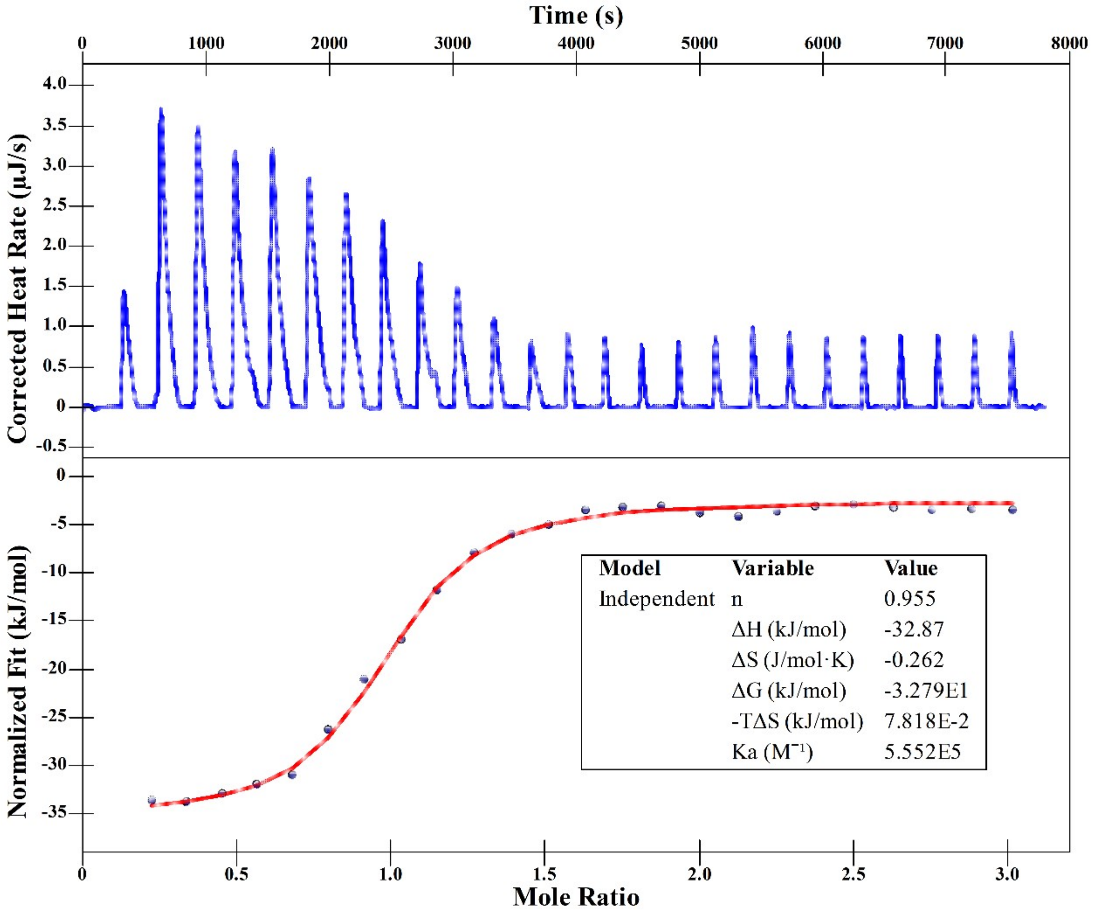

Figure S12. Titration diagram for isothermal titration calorimetry of G3@TMeQ[6].

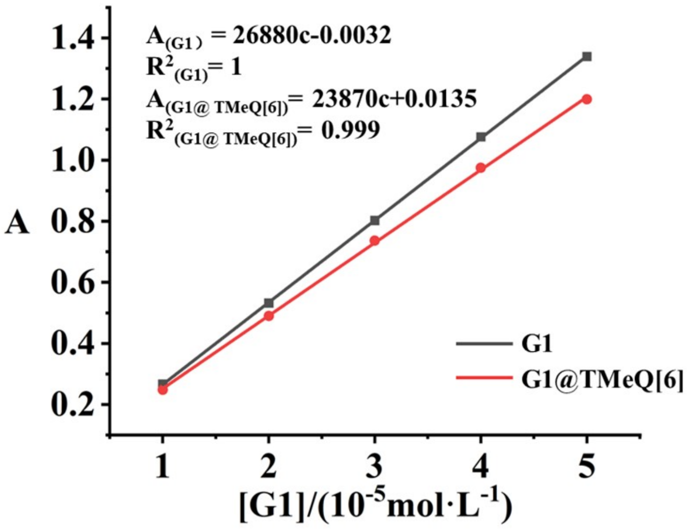

Figure S13. Calibration curves of G1 and G1@TMeQ[6].

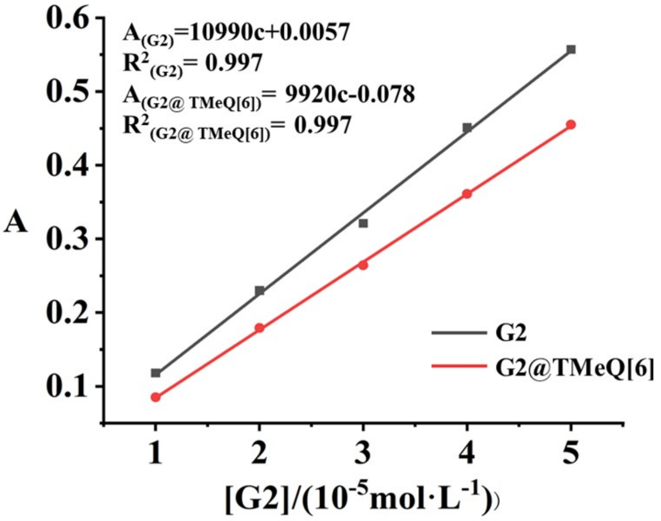

Figure S14. Calibration curves of G2 and G2@TMeQ[6].

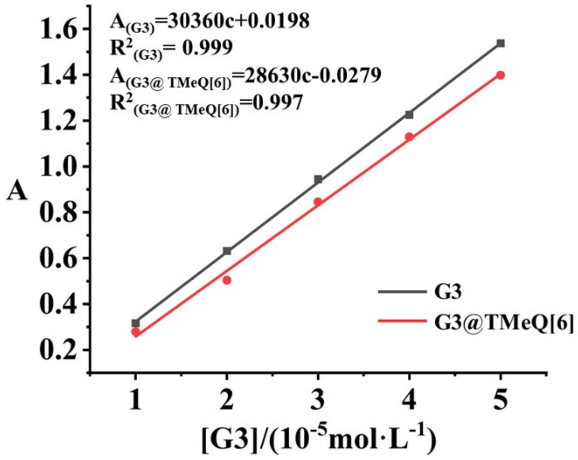

Figure S15. Calibration curves of G3 and G3@TMeQ[6].

Table S2 Crystal structure parameters of complexes **1–3**

Complex	**1**	**2**	**3**
Formula	$C_{54.06}H_{75.12}Cd_2Cl_8N_{28.04}O_{22}$	$C_{52}H_{59}N_{27}O_{12}$	$C_{106}H_{108}Cl_{12}N_{52}O_{24}Zn_3$
M_r	1978.15	1254.26	3115.95
Crystal system	monoclinic	monoclinic	triclinic
Space group	$P2_1/c$	*C*2	*P*-1
a (Å)	12.9500(6)	23.088(6)	13.5914(10)
b (Å)	20.3971(11)	11.877(3)	17.2592(12)
c (Å)	15.0471(7)	15.988(7)	33.599(2)

α (deg)	90	90	82.721(2)
β (deg)	100.3790(10)	131.526(6)	79.158(2)
γ (deg)	90	90	72.244(2)
V [Å^3]	3909.5(3)	3284.4(19)	7351.9(9)
Z	2	2	2
Dc (g cm^{-3})	1.680	1.268	1.408
F(000)	2007	1312	3188
μ (mm^{-1})	0.906	0.095	0.784
Parameters	733	424	1751
θ(deg)	1.700-26.450	3.143-24.999	2.286-25.000
R_{int}	0.0554	0.1518	0.0618
$R[I > 2\sigma(I)]$[a]	0.0797	0.1098	0.1082
$wR[I > 2\sigma(I)]$[b]	0.2280	0.2746	0.3069
R(all data)	0.0924	0.1768	0.1579
wR(all data)	0.2375	0.3182	0.3315
GOF (F^2)	1.136	1.057	1.043
CCDC	2259636	2280674	2278652

[a] Conventional R on Fhkl: $\sum||F_o| - |F_c||/\sum|F_o|$.
[b] Weighted R on $|Fhkl|^2$: $\sum[w(F_o^2 - F_c^2)^2]/\sum[w(F_o^2)^2]^{1/2}$.

We made several attempts to obtain better quality data for this structure however, due to twinning, disorder, poor crystal quality etc. the R_{int} of complex **2** value is high. This structure was included for comparison with the other similar complexes and characterized by ^{1}H NMR spectra. Moreover, data completeness of is 99.4% which guarantees a correct structural elucidation of complex **2**. We are confident the structural characterization is valid. and there is a large amount of disorder in the structure, which caused the level B alerts.

References

[1] H. A. Benesi, J. H. Hildebrand, *J. Am. Chem. Soc.* **1949**, 71, 2703–2707
